# The impact of preoperative patient characteristics on the cost-effectiveness of total hip replacement: a cohort study

**DOI:** 10.1186/1472-6963-14-342

**Published:** 2014-08-15

**Authors:** Matthias Vogl, Rainer Wilkesmann, Christian Lausmann, Werner Plötz

**Affiliations:** Helmholtz Zentrum München, German Research Center for Environmental Health, Institute of Health Economics and Health Care Management, P.O. Box 1129, Neuherberg, 85758 Germany; Ludwig-Maximilians-Universität München, Munich School of Management, Institute of Health Economics and Health Care Management & Munich Center of Health Sciences, Munich, Germany; Department of Orthopaedics and Trauma Surgery, Krankenhaus Barmherzige Brüder München, Akademisches Lehrkrankenhaus der Technischen Universität München, Romanstraße 93, Munich, 80639 Germany; Klinikum rechts der Isar, Technical University Munich, Munich, Germany

**Keywords:** Health-related quality of life, Costs, Cost-effectiveness, Cost-utility, QALY, EQ-5D, WOMAC, Total hip replacement, Hip

## Abstract

**Background:**

To facilitate the discussion on the increasing number of total hip replacements (THR) and their effectiveness, we apply a joint evaluation of hospital case costs and health outcomes at the patient level to enable comparative effectiveness research (CER) based on the preoperative health state.

**Methods:**

In 2012, 292 patients from a German orthopedic hospital participated in health state evaluation before and 6 months after THR, where health-related quality of life (HRQoL) and disease specific pain and dysfunction were analyzed using EQ-5D and WOMAC scores. Costs were measured with a patient-based DRG costing scheme in a prospective observation of a cohort. Costs per quality-adjusted life year (QALY) were calculated based on the preoperative WOMAC score, as preoperative health states were found to be the best predictors of QALY gains in multivariate linear regressions.

**Results:**

Mean inpatient costs of THR were 6,310 Euros for primary replacement and 7,730 Euros for inpatient lifetime costs including revisions. QALYs gained using the U.K. population preference-weighted index were 5.95. Lifetime costs per QALY were 1,300 Euros.

**Conclusions:**

The WOMAC score and the EQ-5D score before operation were the most important predictors of QALY gains. The poorer the WOMAC score or the EQ-5D score before operation, the higher the patient benefit. Costs per QALY were far below common thresholds in all preoperative utility score groups and with all underlying calculation methodologies.

## Background

Because of scarce resources in health care systems and a concurrent increasing number of total hip replacements (THR) worldwide, cost-effectiveness and quality performance measures for THR are becoming more important [1]. With 295 THR per 100,000 inhabitants, Germany currently has the highest THR rate among European Union countries [2]. At over 210,000 operations, THR was among the top 10 surgeries performed in Germany in 2011 [3]. With an aging society, these numbers – and especially the number of revision THR – will increase further. Future supply, development, and innovation in THR will depend more and more on its cost-effectiveness for certain patient groups [4]. Some National Health Service (NHS) trusts in the UK already prioritize patient groups based on patient-reported outcome measures (PROMs) to facilitate efficient and effective THR treatment [5, 6]. The intense discussion on the necessity for and the benefit of the increasing number of THRs can be facilitated by this recent empirical evidence on the average cost-effectiveness of THR in Germany, which has not been analyzed to date. Thus, we apply a joint evaluation of hospital costs and health outcomes at the patient level to enable comparative effectiveness research (CER) based on the preoperative health state. CER provides medical and economic decision support in health care systems with scarce resources.

Currently, there are only separate analyses on the impact of preoperative characteristics on length of stay [7], Health-related Quality of Life (HRQoL) [8–17], and single THR cost studies [18, 19]. Thus, the existing literature informs only on the overall costs and general resource use of THR patients [20, 21]. Because of the high cost and health outcome differences in an international comparison of THR, a patient group specific cost-effectiveness analysis is necessary [19, 22]. Thus, we calculate quality-adjusted life years (QALYs) for several preoperative WOMAC states. QALYs report the number of years gained in perfect health by measuring the difference in HRQoL before and after THR. For reasons of comparability and country specificity, we use three different concepts of cost-effectiveness analyses from patient-reported health states: (1) a QALY calculation based on the UK population preference-weighted index to enable comparison with other studies; (2) a QALY-like calculation based on the German population experience-weighted index (EB-QALY) to introduce the perspective of the general German population; and (3) a visual analog scale adjusted life year calculation (VAS-AL) to use the gold standard of health outcome measures – the VAS – in cost-effectiveness analyses. With an activity-based allocation of costs calculated for each study patient, we calculate the cost utility of THR for each patient.

QALYs are used increasingly by policy supporting institutions such as the National Institute for Health and Care Excellence (NICE) to facilitate decision making [23, 24]. With a CER based on the impact of preoperative patient characteristics on QALYs, we support prioritization and payment by results approaches in health policy and modeling approaches in research. Management interventions to provide cost-effective THR are supported by detailed costing related to preoperative patient characteristics. Thus, the aim of the study is to find preoperative patient characteristics that predict the cost-effectiveness of THR and quantify its impact on cost-effectiveness to support medical decision making based on preoperative patient groups.

## Methods

### Study population

From January to June 2012, 393 patients were eligible to participate, 387 THR patients participated in the baseline health state evaluation and 321 (82%) patients participated in the follow-up. For 292 patients (74%) we received full HRQoL information including all WOMAC and EQ-5D health measures and attributable, patient-based costing measures. Except for lack of patient consent, we had no exclusion criteria. The study was performed at an orthopedic hospital in Munich (Hospital Barmherzige Brüder München, university teaching hospital) and had approval from the ethics committee of Klinikum rechts der Isar, Technical University Munich. All patients followed a similar clinical pathway, which is independent of preoperative patient characteristics, to fulfill the prerequisite of the prognostic study design for patient-group specific information on costs per QALY [25]. For most patients osteoarthritis was the major diagnosis (95.5%); some had osteonecrosis, mechanical complications, or infections that led to THR as the major diagnosis. For 21 patients we had some missing data in covariates. Diagnostic tests showed that we can afford loosing those cases for regression analyses. As imputation could induce another bias in the analysis, we kept the reduced dataset for regression and worked with the full dataset in cost- and effectiveness analyses where a comprehensive set of covariates is not necessary.

### Study design

The innovation in the study design is a detailed acquisition and combination of costs and HRQoL data at the patient level. While we measure costs with a standardized, patient-based DRG costing scheme [26], effectiveness is measured with a generic HRQoL instrument on five health dimensions (EQ-5D) [27] before (baseline) and 6 month after THR (follow-up) to enable change score models that are related to case-based costs and preoperative patient characteristics. In this single center, prospective observation of a cohort, we define a set of preoperative patient characteristics with an impact on HRQoL or costs in prior clinical studies [8–17] and shed light on THR cost-effectiveness for patients with different preoperative characteristics.

To calculate life expectancy, we used the life expectancy of each THR patient based on age and gender from the life tables of the federal statistical office [28] and multiplied them by the EQ-5D change scores to calculate QALYs, assuming that the change score remains constant during each patient’s individual life expectancy. Based on literature recommendations, we discounted QALYs and costs of potential revision by 3.5% [29]. On account of very low differences in HRQoL comparing primary and revision THR [30, 31], and presumed similarity in the literature [5], we did not distinguish between primary and revision THR concerning HRQoL. Based on literature, we assigned a THR revision rate of 7.5% for the first 10 years and 1.5% for each following year [5, 32, 33].

We calculate the average cost-effectiveness ratio (ACER) for several THR groups compared with no intervention. In the medical literature, the ACER and the incremental cost-effectiveness ratio (ICER) are sometimes used interchangeably with each other, especially when the alternative to a procedure is only no intervention [21]. In familiar interventions such as THR, where the alternative is usually no intervention, empirical studies have shown that the ICER is not preferable to the ACER for medical decision making [34]. Thus, all cost-effectiveness ratios in this paper refer to ACER.

### Measuring instruments

Clinical practice guidance mentions reduced HRQoL as a limitation related to osteoarthritis [35]. The EQ-5D is a generic HRQoL instrument, which generates an index value based on a formula with respect to the preferences and experiences of a country’s population out of the five dimensions of mobility, self-care, usual activity, pain/discomfort, and anxiety/depression [27]. A 5-digit code made up of these five domains (each with 3 possible values: no problems, some problems, severe problems) allows 243 health states, which are evaluated based on three different methodologies to allow for comparability with U.K. and U.S. studies and concurrent country specificity [29]. Thereby the best health state is 1,1,1,1,1 (no problems in each dimension) and the worst health state is 3,3,3,3,3 (severe problems in each dimension). The U.K. population preference-weighted index uses a value for each 5-digit code that is based on the time trade-off (TTO) preferences of the general U.K. population [36]. The German population experience-weighted index uses the VAS results from the general German population to allocate a value to each 5-digit state [27]. Additionally, the overall health state is measured using the VAS, which retrieves the overall health state on a 0–100 scale in the study population. The German experience-weighted index and the VAS do not fully qualify for QALY calculation as they are not based on TTO and do not include health states worse than death. Thus, they do not conform to the common QALY definitions and are therefore called experience-based QALY (EB-QALY), when based on the German experience-weighted index, and VAS adjusted life years (VAS-AL), when based on the VAS scale. All EQ-5D calculations allow a comprehensive evaluation of the actual value of an intervention for the patient: it is a comparative effectiveness measure for HRQoL that is recognized as reliable, valid, and responsive in the literature [37, 38]. The WOMAC score is a disease specific score that measures pain and dysfunction of the hip. To measure hip-specific outcome from a patients’ perspective, we use the WOMAC with its three subscales on pain (5 sub-questions), stiffness (2 sub-questions), mobility (17 sub-questions), and an overall score based on the three subscales. To make the overall score comparable to EQ-5D results, we transformed WOMAC results to a 0–100 scale where 0 is the poorest measure and 100 is the best measure. Each WOMAC question has a Likert scale from 0 to 10. The WOMAC shows the best psychometric characteristics in THR specific questionnaires [39–41].

The costing scheme of the Institute for the Hospital Remuneration System (InEK) was introduced to calculate reimbursement rates for DRGs in a transparent and efficient way [42]. In 2011, 263 hospitals participated in the calculation [26]. It provides a detailed, patient-based cost accounting scheme, which is used by hospitals to participate in the reporting process that their reimbursement is based on. We use the InEK costing scheme to calculate case costs and analyze the impact of preoperative characteristics on resource utilization and case costs. Costs and resource use in the scheme are split into several cost-categories (physicians, nursing, medical/technical staff, drugs, implants, other material, medical- and nonmedical infrastructure) and cost-centers (ward, operating room, anesthesia, intensive care, diagnostics/therapy, radiology, and laboratories). The cost breakdown allows a comparison with other THR costing studies in the U.S., the U.K., and other European countries [18, 19, 22].

### Statistical analysis

Patient characteristics with potential impact on cost-effectiveness are included in three subdomains: (1) *socio-demographic factors*, including age, gender, marital status, housing situation, kind of discharge, and kind of insurance; (2) *medical factors*, including BMI, major diagnosis, all frequent secondary diagnoses, the number of secondary diagnoses, ASA classification, Charlson Comorbidity Index, metabolic syndrome, all major procedures and frequent side procedures (e.g., use of cement), number of operations and procedures, number of operations on affected joint before hospital stay, major hip distortion, and preoperative hemoglobin value; (3) *HRQoL, pain, function, and mobility before THR*, measured by EQ-5D, WOMAC, and Harris Hip Score.

Spearman’s correlation was used to examine the bivariate relation of independent variables to costs and QALYs. Covariates with a relation (p < 0.05) were included in a multivariate linear regression analysis (OLS). OLS was used for cost and QALY analyses, as both were normal distributed. A backward selection method was used to determine the final set of covariates in the OLS on costs and QALYs. Covariates were considered relevant for p < 0.05. We provide descriptive statistics for cost and QALY gains separated by preoperative WOMAC groups, the most predictive preoperative patient characteristic according to OLS analysis. For patient differentiation we used the WOMAC as a standardized and specific, patient-based reporting measure. As WOMAC and EQ-5D are correlated we could not eliminate the “regression to the mean” issue [43], a general limitation of cohort studies when analyzing subgroups. Data analysis was performed using SPSS 21 software.

## Results

Some 58% of the study population was female, the average age was 68 years, and most patients had an ASA score of 1 or 2 and a Charlson Comorbidity Index of 0 or 1 (Table 1). Most patients received cementless primary THR with osteoarthrosis of the hip as the major diagnosis. Compared to the population in a large European Study that includes also a representative German sample [44], our population is slightly younger, has less secondary diagnoses (lower average Charlson Comorbidity Index), and patients have shorter length of stay and lower costs. 89% of patients were in the major DRGs for THR (I47A and I47B). Table 1 shows all the control variables and their correlation with costs and QALYs. Correlations were detected, e.g., for acute anemia, diabetes, depression, sleep disorder, major hip distortion, number of secondary diagnoses, number of operation and procedure codes, open reposition fracture, operations at joint before procedure, housing situation, health insurance, ASA score, and Charlson Comorbidity Index.

**Table 1:** **Impact of relevant preoperative patient characteristics**

Significant Spearman’s rank correlation: relation of secondary diagnoses, operations/procedures, and further parameters with *costs and ^QALYs (p < 0.05)	Frequency/mean (SD)	%	Missing
**Secondary diagnoses, at least 10 times**			
D62 - Acute anemia*	26	8.9	1
E03 - Hypothyroidism	48	16.5	1
E11 - Diabetes*	23	7.9	1
E66 - Obesity	18	6.2	1
E78 - Lipidemia	30	10.3	1
E79 - Purine/pyrimidine metabolism	13	4.5	1
E86 - Hypovolemia*	13	4.5	1
E87 - Dysfunction of water/electrolyte balance*	18	6.2	1
F32 - Depression^	12	4.1	1
G47 - Sleep disorder*	10	3.4	1
I10 - Arterial hypertonicity	160	55.0	1
I25 - Ischemic heart disease	16	5.5	1
I48 - Atrial fibrillation	12	4.1	1
J45 - Asthma^	12	4.1	1
M16 - Coxarthrosis	281	96.6	1
N18/N39 - renal failure and related diseases	17	5.8	1
T81 - Other complications*	11	3.8	1
Z86 - Personal anamnesis other diseases	11	3.8	1
Z88 - Drug allergy	15	5.2	1
Z91 - Risk factors in personal anamnesis	15	5.2	1
Z92 - Care of personal anamnesis	43	14.8	1
Z95 - Cardiac/vascular implants	24	8.2	1
Z96 - Other functional implants	48	16.5	1
n.n. - Cardiopathy	28	9.6	0
n.n. - COPD	12	4.1	0
n.n. - Hypercholesterolemia	44	15.1	0
n.n. - Myocardial infarction/stent	20	6.9	0
n.n. - Reflux^	18	6.2	0
n.n. - Major hip distortion*^	20	7.0	0
n.n. - deep venous thrombosis (DVT)	12	4.1	0
Number of secondary diagnoses*	3.4 (3.0)		1
**Operations and procedures, at least 5 times**			
5-782 - Excision/resection of diseased bone*	5	1.7	2
5-791 - Open reposition of fracture*	6	2.1	2
5-820 - Primary endoprosthesis (no revision)	281	96.7	2
5-821 - Revision*	10	3.4	2
5-829 - Other arthroplasty*	10	3.4	2
5-986 - Minimally invasive technique	271	93.4	2
8-919 - Acute pain relief	5	1.7	2
8-930/1 - Monitoring*	30	10.3	2
Number of operation and procedure codes*	2.4 (1.2)		2
**Other**			
Age^	68 (10.2)		1
Gender male	121	41.6	1
BMI*	26.8 (4.9)		7
≥30	49	17.2	
Major diagnosis*			1
Osteoarthritis	279	95.5	
Osteonecrosis	3	1.0	
Mechanical complications/infections	9	3.1	
Operations at joint before procedure*^			1
0	265	91.1	
1	19	6.5	
2 or more	7	2.4	
Marital status			3
Married	192	66.4	
Single	25	8.7	
Divorced/living apart	34	11.8	
Widowed	38	13.1	
Housing situation			4
Alone	81	28.1	
With partner	146	50.7	
With family	61	21.2	
Discharge home (other, inpatient rehabilitation)	56	19.2	3
Health insurance compulsory (other, private)*	149	51.2	1
Already THR	61	20.9	0
Preoperative hemoglobin*	14.0 (1.2)		0
Blood transfusion/erythrocyte concentrate*	16	5.5	2
ASA score*			0
1	110	37.7	
2	158	54.1	
3 or higher	24	8.2	
Charlson Comorbidity Index*			1
0	217	74.6	
1	57	19.6	
2	10	3.4	
3 or higher	7	2.4	
Metabolic syndrome*	8	2.7	0
Cement or hybrid (other, cementless)^	34	11.9	7

OLS regressions showed the relationship of several preoperative characteristics to inpatient costs and QALYs. The impact of WOMAC and EQ-5D was provided separately to avoid multi-collinearity. Both preoperative measures are correlated. For the grouping of QALY gains we used the WOMAC as it is most accepted among orthopedics and mostly used in day-to-day routines. Both preoperative scores have similar impact on QALY gains but low impact on inpatient costs, as patients were treated very similar. Generally, there was low variance in inpatient THR costs but the number of operation and procedure codes, the number of secondary diagnoses, health insurance, and revision THR had an impact on costs (Table 1). Mean costs of THR were € 6,310 (95% confidence interval € 6,160 - €6,472) with the highest costs for ward and operating room in the cost-center view and the highest costs for physicians, implants, and infrastructure in the cost category view (Table 2). Revision THR increases inpatient costs by 18%. Average life expectancy of the study population is 17.57 years. Studies show that the probability of revision is 7.5% for the first 10 years after primary THR and 1.5% for each year thereafter [5, 33], resulting in average lifetime inpatient costs of € 7,730 for THR. The grouping by preoperative WOMAC score shows a trend that healthier patients cost less (Table 3).

**Table 2:** **Inpatient cost calculation**

Cost matrix*	Physicians	Nursing	Medical/technical staff	Drugs general	Drugs individual	Implants and grafts	Material	Material individual	Medical infrastructure	Non-medical infrastructure	All cost categories
Ward	438	731	0	64	1	0	53	3	196	765	**2,251**
Intensive care	7	18	0	3	0	0	4	0	1	6	**40**
Operating room	434	0	306	6	0	1,709	165	64	162	229	**3,075**
Anesthesia	242	0	153	22	0	0	49	0	29	69	**563**
Cardiac diagnostics	1	0	0	0	0	0	0	9	0	0	**10**
Endoscopic diagnostics	1	0	1	0	0	0	1	0	1	1	**4**
Radiology	22	0	26	4	0	0	8	4	6	22	**91**
Laboratories	2	0	36	6	11	0	32	8	4	12	**111**
Further diagnostics/therapy	2	0	113	0	0	0	3	0	4	44	**166**
**All cost-centers**	**1,148**	**749**	**635**	**104**	**12**	**1,709**	**314**	**87**	**403**	**1,148**	**6,310**

*differences in the totals fields are due to rounding errors.

**Table 3:** **QALY calculation – separated by preoperative WOMAC score**

Visual analog scale (gold standard): VAS adjusted life years (VAS-AL)																		
WOMAC pre-operative	N	Mean EQ-5D VAS pre-operative	SD	Mean EQ-5D VAS post-operative	SD	Mean change EQ-5D VAS	SD	Life expectancy	SD	Mean costs in €	SD	Mean costs incl. potential revision	SD	VAS-AL	SD	VAS-AL disc. 3.5%	Cost/VAS-AL in €	Cost/VAS-AL disc. 3.5%
0-20	14	47.50	21.68	74.86	16.49	27.36	23.26	16.48	7.95	7,043	1,813	8,421	1,778	4.10	3.25	3.38	2,055	2,313
21-40	75	50.09	22.85	71.99	22.07	21.89	27.63	16.71	8.68	6,698	1,860	8,100	2,304	4.46	6.93	2.73	1,816	2,737
41-60	110	59.19	17.86	78.10	19.95	18.91	22.82	17.16	7.9	6,198	1,036	7,582	1,843	3.25	4.88	2.41	2,332	2,892
61-80	77	68.09	16.48	83.31	12.44	15.22	20.35	18.58	7.16	6,034	978	7,487	1,419	2.79	3.99	2.05	2,684	3,310
81-100	16	79.69	16.23	86.63	10.79	6.94	17.55	20.39	7.61	5,948	617	7,568	1,118	2.27	4.73	1.00	3,337	6,757
**Sum**	**292**	**59.76**	**20.72**	**78.22**	**18.78**	**18.45**	**23.55**	**17.57**	**7.92**	**6,310**	**1,341**	**7,730**	**1,854**	**3.43**	**5.23**	**2.39**	**2,256**	**2,964**
**UK preference-weighted index as underlying value set (QALY)**																		
**WOMAC pre-operative**	**N**	**Mean EQ-5D utility score pre-operative**	**SD**	**Mean EQ-5D utility score post-operative**	**SD**	**Mean change EQ-5D utility score**	**SD**	**Life expectancy**	**SD**	**Mean costs in €**	**SD**	**Mean costs incl. potential revision**	**SD**	**QALY**	**SD**	**QALY disc. 3.5%**	**Cost/QALY in €**	**Cost/QALY disc. 3.5%**
0-20	14	0.04	0.12	0.81	0.17	0.77	0.16	16.48	7.95	7,043	1,813	8,421	1,778	12.40	6.17	9.50	679	823
21-40	75	0.30	0.31	0.76	0.25	0.46	0.33	16.71	8.68	6,698	1,860	8,100	2,304	8.20	8.78	5.76	987	1,300
41-60	110	0.54	0.25	0.86	0.15	0.31	0.28	17.16	7.90	6,198	1,036	7,582	1,843	5.85	6.77	3.98	1,295	1,751
61-80	77	0.70	0.17	0.88	0.16	0.18	0.24	18.58	7.16	6,034	978	7,487	1,419	3.28	4.64	2.40	2,281	2,829
81-100	16	0.80	0.06	0.94	0.10	0.14	0.14	20.39	7.61	5,948	617	7,568	1,118	3.16	2.81	2.03	2,393	3,329
**Sum**	**292**	**0.51**	**0.30**	**0.84**	**0.19**	**0.33**	**0.31**	**17.57**	**7.92**	**6,310**	**1,341**	**7,730**	**1,854**	**5.95**	**7.08**	**4.25**	**1,300**	**1,669**
**German experience-weighted index as underlying value set (EB-QALY)**																		
**WOMAC pre-operative**	**N**	**Mean EQ-5D utility score pre-operative**	**SD**	**Mean EQ-5D utility score post-operative**	**SD**	**Mean change EQ-5D utility score**	**SD**	**Life expectancy**	**SD**	**Mean costs in €**	**SD**	**Mean costs incl. potential revision**	**SD**	**EB-QALY**	**SD**	**EB-QALY disc. 3.5%**	**Cost/EB-QALY in €**	**Cost/EB-QALY disc. 3.5%**
0-20	14	0.34	0.06	0.72	0.17	0.39	0.15	16.48	7.95	7,043	1,813	8,421	1,778	6.49	4.60	4.76	1,297	1,643
21-40	75	0.44	0.13	0.71	0.18	0.27	0.18	16.71	8.68	6,698	1,860	8,100	2,304	4.71	5.09	3.39	1,721	2,207
41-60	110	0.53	0.12	0.77	0.14	0.24	0.17	17.16	7.90	6,198	1,036	7,582	1,843	4.39	4.17	3.04	1,726	2,292
61-80	77	0.65	0.12	0.80	0.12	0.15	0.17	18.58	7.16	6,034	978	7,487	1,419	2.79	3.62	2.02	2,685	3,368
81-100	16	0.73	0.08	0.86	0.06	0.12	0.11	20.39	7.61	5,948	617	7,568	1,118	2.59	2.20	1.75	2,920	3,856
**Sum**	**292**	**0.54**	**0.16**	**0.76**	**0.15**	**0.22**	**0.18**	**17.57**	**7.92**	**6,310**	**1,341**	**7,730**	**1,854**	**4.05**	**4.32**	**2.90**	**1,908**	**2,439**

Mean EQ-5D utility score change was 0.33 with the U.K. preference based value set as the underlying utility scores (preoperative 0.51; postoperative 0.84). With the German experience-based value set, EQ-5D utility score change was 0.22 (preoperative 0.54; postoperative 0.76). The VAS score change was 18.45 (preoperative 59.76; postoperative 78.22). Change scores were not affected by either gender or age. Bivariate analysis showed high correlation of preoperative WOMAC scores with QALYs gained. In a multivariate OLS analysis, the preoperative EQ-5D and WOMAC scores had the highest impact on the effectiveness of THR and therefore QALYs (Table 4). Thus, we provided QALY calculation for preoperative WOMAC groups. Among all preoperative characteristics, the preoperative WOMAC score suits best for payment by results approaches, modeling approaches, and management interventions to provide cost-effective THR.

**Table 4:** **Multivariate regression analysis on costs and QALY gains**

Dependent variable: QALYs (U.K. preference-weighted)	Coefficient	Beta	SE	p-value
Constant	43.810		3.130	.000
*Preoperative WOMAC sum*	-.153	-.396	.018	.000
ICD F32 - Depression	−3.266	-.096	1.597	.042
G47 - Sleep disorder	−4.843	-.109	2.327	.038
Reflux	−2.645	-.094	1.337	.049
Major hip distortion	−3.770	-.140	1.290	.004
OPS 8-930/1 - Monitoring	2.709	.107	1.334	.043
Age	-.331	-.466	.034	.000
n = 271 Adj. R^2^ = .418				
**Dependent variable: QALYs (U.K. preference-weighted)**	**Coefficient**	**Beta**	**SE**	**p-value**
Constant	42.268		2.240	.000
*Preoperative EQ-5D utility score (U.K. preference-weighted)*	−15.316	-.658	.805	.000
ICD F32 - Depression	−2.673	-.078	1.176	.024
Reflux	−1.829	-.065	.970	.060
Major hip distortion	−3.205	-.119	.946	.001
Age	-.318	-.447	.026	.000
ASA 2 (compared to ASA 1)	-.846	-.060	.498	.090
n = 271 Adj. R^2^ = .685				
**Dependent variable: inpatient costs**	**Coefficient**	**Beta**	**SE**	**p-value**
Constant	6,338		845	.000
Preoperative EQ-5D utility score (U.K. preference-weighted)	−426	-.109	177	.017
ICD J45 - Asthma	−508	-.085	274	.065
Number of secondary diagnoses	103	.242	25	.000
OPS 5–782 - Excision/resection of diseased bone	1,435	.147	468	.002
OPS 5–791 - Open reposition of fracture	1,699	.194	401	.000
OPS 5–821 - Revision	1,811	.185	462	.000
OPS 8-930/1 - Monitoring	−686	-.162	233	.004
Number of operations and procedures	461	.409	63	.000
Age	−11	-.098	5	.038
BMI	25	.105	10	.018
Preoperative hemoglobin	−83	-.081	47	.079
n = 271 Adj. R^2^ = .502				

**Figure 1:**
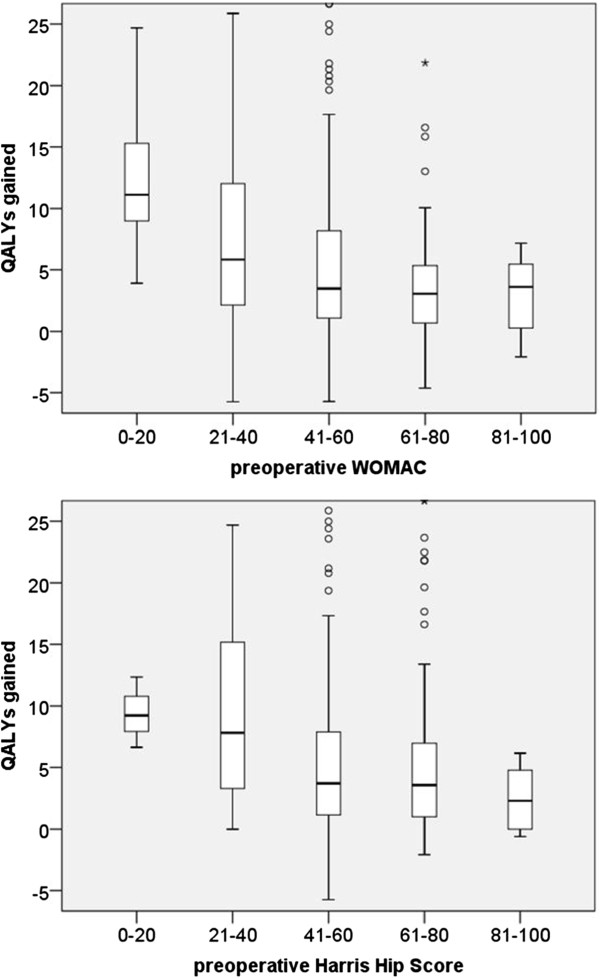
**QALYs by preoperative WOMAC and harris hip score.**

QALYs gained using the U.K. preference based value set were 5.95. Lifetime costs per QALY were € 1,300. Using the German experience-based value set as the underlying set for EQ-5D utility scores showed an EB-QALY gain of 4.05 with € 1,908 per EB-QALY. The VAS showed a VAS-AL gain of 3.43 and costs of € 2,256 per VAS-AL. VAS results were divided by 100 to calculate VAS-AL and to be comparable with the U.K. QALYs and the German EB-QALYs. All three value sets for QALY and QALY-like calculation showed a large increase in QALY gains with a decrease in preoperative WOMAC scores, indicating that patients with poor WOMAC scores benefit most from THR (Table 3). QALY gains are higher when patients are less healthy. The separation of study participants into age groups showed that even the oldest age group, which naturally gains the fewest QALYs, had low QALY costs (Table 5). The economic perspective of QALYs is further correlated with direct medical pain and dysfunction scores, e.g., the Harris Hip Score (Figure 1), showing that physician based questionnaires can also be used for a health economics perspective.

**Table 5:** **QALY gains by age**

Underlying value set	Age group			
	≤59	60-69	70-79	≥80
	**QALYs**			
VAS-AL based	6.398	3.280	1.697	0.990
UK QALY based	10.967	6.122	4.341	2.531
German EB-QALY based	7.565	4.098	2.583	1.506
	**QALYs discounted 3.5%**			
VAS-AL based	3.922	2.357	1.953	0.860
UK QALY based	6.723	4.399	3.105	2.199
German EB-QALY based	4.637	2.944	2.269	1.308
	**Cost/QALY in €**			
VAS-AL based	1.927	3.085	3.771	9.391
UK QALY based	1.124	1.653	2.372	3.672
German EB-QALY based	1.630	2.469	3.246	6.171
	**Cost/QALY in €; discounted 3.5%**			
VAS-AL based	1.738	2.857	3.569	9.071
UK QALY based	1.014	1.530	2.245	3.547
German EB-QALY based	1.470	2.286	3.072	5.961

## Discussion

Multivariate regression analyses gave an overview of the actual drivers of cost-effectiveness in THR: costs were mainly increased by special procedures, such as excision/resection of the diseased bone and open reposition of fracture, or by revision (Table 4). Preoperative patient characteristics had no important and systematic impact on THR costs. On the effect side, some preoperative characteristics can impact health outcome significantly. Depression, reflux, and major hip distortion significantly reduce QALY gains (Table 4). A higher ASA score and revision compared with primary THR also significantly reduce QALY gains. Increased age and high EQ-5D scores reduce QALY gains the most. We have average THR costs of € 6,310 with a 95% confidence interval of € 6,160 - €6,472, with a trend that healthier patients cost less (Table 3). In countries with similar purchasing powers to those in the U.S. or Europe – and where health insurance is available – costs are not the limiting factor when contrasting them with QALY gains in cost-effectiveness analyses. Patient characteristics that affect QALY gains the most were age and preoperative WOMAC (or EQ-5D) score, which qualifies these parameters to be included in shared decision making with the patient for professionals. This makes a QALY threshold, but not a cost per QALY threshold, important for health policy related to THR.

As the WOMAC score and EQ-5D scores predict the patient benefit and QALY gains best, they should be used in patient reported outcome measures (PROMs) in the future. PROMs are becoming important as a quality measure from the patient’s perspective and for pay for performance approaches. Because of the differentiation by preoperative WOMAC, results can support patients in their decisions on THR, they can support health policy with prioritization decisions based on QALY thresholds and net benefit analysis based on willingness to pay, and they can support hospital management through a differentiated analysis of costs (Table 2) and the impact of patient and care selection on costs and QALY gains (Table 4). Age is the second driver of QALY gains. Similar to other studies on THR cost-effectiveness [33, 45], age has a low impact on the EQ-5D change score itself (younger people expect slightly higher health benefits from THR), but it affects the life expectancy component of QALY calculation. Still, all WOMAC and age groups benefit from THR and have low costs per QALY (Tables 3 and 5). THR is a cost-effective intervention for all analyzed WOMAC and age groups when applying the common cost per QALY thresholds from NICE, the World Health Organization, and other institutions that provide national guidance and advice on improving health care [21, 46].

The advantages of ACER analysis in this study, according to Bang and Zhao [47], are that (1) clinical and economical characteristics of THR are analyzed independently of an alternative or no intervention; (2) ACER results are thus intuitive for research and policy; (3) results are more robust compared with ICER; and (4) a subjective cost per QALY threshold is not needed for decision making. Further advantages of the study were, first, the detailed bottom-up costing approach for each patient that was based on the actual resource use of each single patient. The costing method is verifiable and comparable, as each case had to pass plausibility checks by the InEK to be part of the study [26]. The separation of costs into components (Table 2) enables comparative and transparent costing analyses. Second, the prospective study design allowed the attribution of actual patient costs to health outcome scores to calculate cost-utility (QALY) for each patient. Thus, it is the first German study to report cost-effectiveness or cost-utility values for THR. The calculation of EB-QALYs and VAS-ALs besides QALYs has the advantage that – although they do not conform to the rigorous QALY definition – EB-QALYs and VAS-ALs are closer to the actual experiences of THR patients than QALYs but still have the advantages of QALY calculation.

Limitations of the study include: (1) inability to retrieve health states after a potential revision or for longer follow-up periods. Thus, we extrapolated the EQ-5D utility score change of the 6-month follow-up for QALY calculation. In this way, we assume that the change score remains constant during each patient’s individual life expectancy. This assumption is rather conservative, as the literature shows that HRQoL for THR patients is slightly higher a year after operation compared with at the 6-month follow-up [48]. However, this method is widely accepted and used in the literature [5, 48]; (2) the EQ-5D change scores of revision patients differed slightly from primary THR change scores (−0.02) in our study. Similar to other studies, we assumed that revisions keep the change in HRQoL at the 6-month follow-up level [5]; (3) 18% of patients were lost in the follow-up. They did not differ significantly in preoperative socio-demographic and medical factors. However, similar to other THR studies [5], they had slightly lower mean preoperative EQ-5D scores (−0.01 with the experience-weighted index), which indicates a conservative estimate and a slightly higher EQ-5D change score if these patients were included in the study. As we have a monocentric study, representativeness of patients might not be given; (4) we used the WOMAC as a preoperative patient characteristic for health state and dysfunction for categorizing QALY results. However, as WOMAC and EQ-5D utility scores are correlated, we could not eliminate the general limitation of regression toward the mean, potentially leading to inaccurate results when comparing preoperative patient groups on costs and QALYs. The use of WOMAC in standardized PROMs is more likely anyway, as disease specific instruments such as the Oxford Hip/Knee Score are already used for prioritization in the U.K. [6]; and (5) the inpatient cost accounting scheme used had some minor limitations [26, 42]. The full-cost approach did not include capital costs, and although German DRG costing has a high accuracy, it is not the gold standard in every calculation step.

In comparison with other cost-effectiveness literature, our results range as follows: average QALY gains were similar to many U.K. and U.S. studies [5, 33, 49, 50]. However, especially in methodologically similar UK studies, average preoperative health scores are lower compared with this study, allowing a higher increase in scores [5]. Here, we suppose an operation at an earlier coxarthrosis stage in Germany than in the U.K. Costs per QALY were mostly similar [5, 33] or lower [20, 21, 49] compared with methodologically comparable studies.

In a future perspective, studies should take a closer look at the actual comparative group of THR patients. In this study, we identified the preoperative WOMAC score as a good predictor of QALY gains. However, similar to most other studies, we compared with a virtual group of patients with no intervention and assumed that their health state remained at the level of THR patients before operation. This might under- or overestimate the real benefit of THR compared with no intervention or compared with pain relief instead of THR [51]. Thus, WOMAC and health outcomes should be analyzed for patients who decided against THR compared with THR patients. A separation into preoperative WOMAC groups, as in this study, can then show which patients benefit most and for which patients cost-utility is highest with conservative therapy. A matching of pre- and postoperative EQ-5D values with population normative values can quantify the utility of total hip replacement for preoperative health state groups. Finally, a comparison of patient reported outcome measures and related micro-costing with other countries allows a detailed comparison of cost-effectiveness and possibly conclusions on weakness in the health care system.

## Conclusions

The WOMAC score and the EQ-5D score before operation were the most important predictors of QALY gains. The poorer the WOMAC score or the EQ-5D score before operation, the higher the patient benefit. Although differences in QALY gains and costs per QALY were high between the three methodologies of cost-utility analysis, they were far below common thresholds in all preoperative utility score groups and with all underlying calculation methodologies [21, 46]. CER results might be used in pay for performance approaches for an efficient and effective health care system [52]. Health outcome and micro-costing measures in combination are useful performance measures to compare hospitals on a patient-level basis. CER results support patients in a shared decision making situation before THR based on a personalized risk assessment approach. They support prioritization decisions in health policy concerning preoperative health states. And they support hospital management through differentiated costing and health outcome measures on a patient basis.

## Abbreviations

ACER: Average cost-effectiveness ratio
ASA: American Society of Anesthesiologists
CER: Comparative effectiveness research
DRG: Diagnosis related group
HRQoL: Health-related quality of life
EB-QALY: Experience-based quality-adjusted life year
EQ-5D: EuroQol 5-dimension
EQ-5D VAS: EuroQol 5-dimension visual analogue scale
ICER: Incremental cost-effectiveness ratio
InEK: Institute for the Hospital Remuneration System
NICE: National Institute of Health and Care Excellence
NHS: National Health Service
OLS: Ordinary least squares
PROMs: Patient-reported outcome measures
THR: Total hip replacement
TTO: Time trade-off
QALY: Quality-adjusted life year
VAS-AL: Visual analogue scale adjusted life years
WOMAC: Western Ontario and McMaster Universities Arthritis Index.
